# Hierarchical clustering for multiple-crystal macromolecular crystallography experiments: the *ccCluster* program

**DOI:** 10.1107/S1600576717015229

**Published:** 2017-11-29

**Authors:** Gianluca Santoni, Ulrich Zander, Christoph Mueller-Dieckmann, Gordon Leonard, Alexander Popov

**Affiliations:** a Structural Biology Group, European Synchrotron Radiation Facility, 71 Avenue des Martyrs, 38000 Grenoble, France; b EMBL Grenoble, 71 Avenue des Martyrs, 38000 Grenoble, CEDEX 9, France

**Keywords:** cluster analysis, serial crystallography, multicrystal data collection, phasing

## Abstract

This article describes *ccCluster*, a software providing an intuitive graphical user interface (GUI) and multiple functions to perform hierarchical cluster analysis on multiple crystallographic datasets. The program makes it easier for users to choose, in the case of multi-crystal data collection, those datasets that will be merged together to give good final statistics. It provides a simple GUI to analyse the dendrogram and various options for automated clustering and data merging.

## Abstract 2

The *ccCluster* program provides an easy-to-use interface to perform hierarchical cluster analysis on protein diffraction datasets.

## 1. Introduction   

The increasing brightness of beamlines for macromolecular crystallography (MX) has been a continuing trend in recent years. This, coupled with the development of single-photon-counting pixel detectors and so-called ‘shutterless’ data collection, has translated into faster data collection and, owing to higher flux densities, the collection of X-ray diffraction data from very small crystals of biological macromolecules. However, because of radiation damage effects, the obtainable resolution of a complete dataset is reduced as the crystal volume becomes smaller. A valuable strategy for overcoming this and the limitations imposed by radiation damage consists of collecting small partial datasets (Garman, 2010 ▸; Owen *et al.*, 2011 ▸) from a series of crystals and merging these to construct a complete dataset. This strategy, known as multi-crystal or serial crystallography, is now commonly practised at X-ray free-electron lasers and synchrotron sources. Two main categories of multi-crystal data collection have been developed: those that rely on the collection of a series of ‘still’ diffraction images from crystals introduced into the X-ray beam using liquid/grease injectors (Chapman *et al.*, 2011 ▸; Nogly *et al.*, 2015 ▸; Botha *et al.*, 2015 ▸) or raster scanning (Coquelle *et al.*, 2015 ▸; Owen *et al.*, 2017 ▸; Roedig *et al.*, 2016 ▸; Oghbaey *et al.*, 2016 ▸); and those where raster scanning is coupled with a rotation of the sample holder, as in some synchrotron serial crystallography (SSX) methods (Zander *et al.*, 2015 ▸; Gati *et al.*, 2014 ▸). Multiple-crystal data collections have also been successfully applied to single-wavelength anomalous diffraction (SAD) phasing (Liu & Hendrickson, 2015 ▸; Olieric *et al.*, 2016 ▸; Weinert *et al.*, 2014 ▸), in particular for native S-SAD, where the anomalous signal level is weak and redundancy of the data becomes fundamental for precise measurement of anomalous differences. Here, since the anomalous differences that are to be measured are rather small, a high level of isomorphism between merged datasets is also essential.

When a few degrees – or more – of oscillation data per crystal are available, diffraction images can be processed by standard crystallographic software such as *XDS* (Kabsch, 2010 ▸) or *DIALS* (Waterman *et al.*, 2013 ▸), and the resulting partial datasets merged to produce the final complete dataset. Here, to achieve the best results, hierarchical cluster analysis (HCA) can be applied to select a suitable subset of the partial datasets for merging. This method, aimed at determining the most isomorphous datasets out of a large number, has already been successfully used (Giordano *et al.*, 2012 ▸; Foadi *et al.*, 2013 ▸). A complementary approach uses global optimization algorithms, such as genetic algorithms (Zander *et al.*, 2016 ▸), to indicate the best grouping of partial datasets in order to achieve the best final statistics possible. Genetic algorithms, however, rely on hundreds of scaling and merging runs, rather than just the few required for HCA, and are thus more time consuming than HCA, often requiring several hours to converge to a result. More recently, a new algorithm has also been published to distinguish between random and systematic errors and account for the case when datasets are highly partial or weak and thus below the limits of application of HCA (Diederichs, 2017 ▸).

In HCA one can use either unit-cell variations (Foadi *et al.*, 2013 ▸) or the correlation coefficients (cc_(*a*,*b*)_) between common intensities in different datasets *a* and *b* (Giordano *et al.*, 2012 ▸) as a metric of non-isomorphism. However, for very small partial datasets unit-cell parameters usually cannot be determined with sufficient accuracy and thus, provided enough partial datasets are available, the use of intensity-based correlation coefficients would seem to be more reliable (Giordano *et al.*, 2012 ▸). Here, we present the software *ccCluster*, the main goals of which are to provide HCA based on cc_(*i*,*j*)_ and to provide a graphical user interface (GUI) making the interpretation of, and interaction with, the resulting dendrogram more accessible to users. A major improvement from the previous implementation is that by using *ccCluster* merging of partial datasets can be directly performed, without manual editing of input files for *XSCALE* (Kabsch, 2010 ▸) or *POINTLESS* (Evans & Murshudov, 2013 ▸), and multiple thresholds can be rapidly tested and compared *via* the software interface to achieve the best final statistics. The tools developed can also be used in automated pipelines for protein structure solution using many partial datasets. *ccCluster* provides both an easy-to-use graphical interface for HCA and a large choice of options for command-line operation. The software is already available for users at the ESRF and can be obtained at http://github.com/gsantoni/ccCluster (http://doi.org/10.5281/zenodo.580254) under the FreeBSD license.

## 2. Software description and theory   

### 2.1. Program and dependencies   

**Figure 1:**
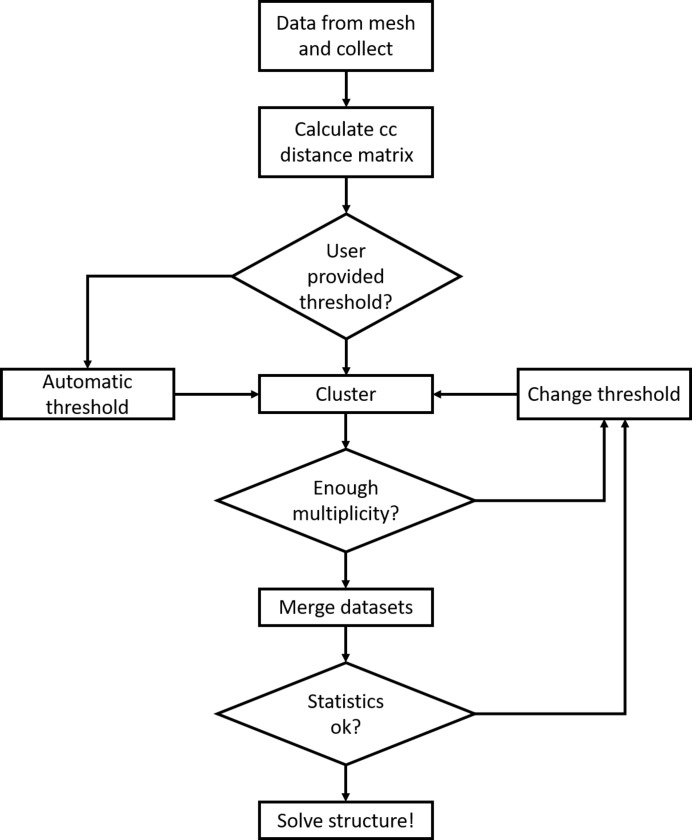
Flowchart of HCA using *ccCluster*. Input files can come from either *XDS* or *DIALS* processing. Merging is performed automatically with *XSCALE*, but a *POINTLESS*–*AIMLESS *run is also possible.

*ccCluster* is written in Python 2.7, using *cctbx* (Grosse-Kunstleve *et al.*, 2002 ▸) for crystallographic data manipulation and *NUMPY* for cluster analysis. The *ccCluster* GUI has been written in PyQt5, using *matplotlib* (Hunter, 2007 ▸). A flowchart of how HCA is implemented within *ccCluster* is presented in Fig. 1 ▸. In the last step of the procedure *ccCluster* calls well established software, in particular *XSCALE* (Kabsch, 2010 ▸) for the merging of partial datasets, and in each output folder produces a simple script allowing users to run the program *POINTLESS* (Evans & Murshudov, 2013 ▸) in order to produce directly an unmerged mtz file. This can then be used by the program *AIMLESS* (Evans & Murshudov, 2013 ▸) to produce reflection data files suitable for downstream processes in *CCP4* (Winn *et al.*, 2011 ▸) and other crystallographic software packages.

### 2.2. Distance matrix calculation and clustering method   

HCA requires a definition of distance between all possible pairs of datasets. The calculation of these distances is performed by the ccCalc class in *ccCluster*. This class has two functions: one for loading all partial datasets to be analysed and the other to calculate the distance between them. The distance, chosen using a command-line option, is defined on the basis of either unit-cell variation or an intensity-based correlation coefficient. For the latter a distance defined by
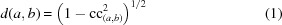
has proven to be suitable for the selection of partial datsets to merge (Giordano *et al.*, 2012 ▸). *ccCluster* uses the same metric, but instead of relying on 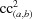 as calculated by *XSCALE* (Kabsch, 2010 ▸), which are calculated after applying corrections to the individual datasets, this is directly obtained using the *cctbx* method miller_array.correlation.coef f icient. Here, the consistency of unit-cell parameters between datasets *a* and *b* is verified with the *cctbx* function assess_symmetry() and 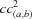 is then calculated from the common reflections in each pair of unmerged datasets. When unit-cell parameters for two datasets are not compatible, *i.e.* when they differ by more than 1%, their distance is assigned a value of 1, corresponding to a null correlation. This procedure helps in the determination of outliers.

As noted above, variation in unit-cell parameters can also be used for HCA of partial datasets in *ccCluster*. Here, inspired by *BLEND* (Foadi *et al.*, 2013 ▸) which uses the variation of the unit-cell diagonal, we calculate the distance between datasets from the maximal variation of one of the unit-cell lengths *A*, *B* or *C*:
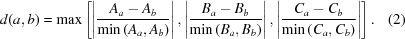
It is, however, important to note that the unit-cell parameters are highly sensitive to detector distance refinement and that not all three parameters are precisely determined when the diffraction wedges have less than 10° rotation. Thus, in *ccCluster* a distance based on 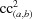 is set as the default option.

The clustering deployed in *ccCluster* uses the average linkage method, which defines the distance between two clusters *X* and *Y* as the average of the distances between all pairs of datasets from the two clusters:
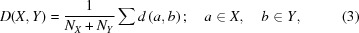

*N*_*X*_ and *N*_*Y*_ being the number of datasets in clusters *X* and *Y*.

### 2.3. Threshold estimation   

As the aim of HCA as implemented in *ccCluster* is to produce a complete dataset by merging many partial datasets, *ccCluster* contains an automatic threshold height determination routine, called ‘minimal for completeness’. Once a dendrogram is generated, this routine concatenates all the reflection files from a cluster at a fixed threshold level and calculates the overall completeness of the resulting Miller array. It then gives an estimation for the minimal value of the threshold at which the dataset is more than 98% complete. The completeness level can be tuned by the user if desired. From its definition [equation (3)], the clustering threshold is directly correlated with the expected average 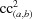 between the merged datasets in the cluster. For example, a clustering at 0.4 will translate to an average 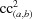 of ∼91% between all the datasets within the selected cluster. Clearly, choosing the lowest threshold possible to obtain the desired dataset completeness should give the highest level of 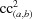 and thus the best merging quality.

When operating from the GUI, the desired threshold height can be changed directly from the dendrogram representation by clicking on the dendrogram itself. This allows users to rapidly perform multiple merging tests, using different threshold levels, in order to achieve optimal merged dataset quality. A simplified threshold estimation is in any case performed when the program is launched, to give the user some idea of an acceptable clustering strategy. This simpler routine, faster than the minimal threshold for completeness, computes the increase in number of datasets in the largest cluster as a function of the threshold. It estimates an adequate clustering threshold, corresponding to the maximum value of this variation.

### 2.4. Merging of partial datasets   

Once a dendrogram has been generated, *ccCluster* performs merging of partial datasets by running the program *XSCALE* in the background. Two options are possible at this step. Either the largest cluster or all clusters below a chosen linkage threshold are merged. Additionally, the user can choose to flag the data as ‘anomalous on’ (Friedel’s law is false) or ‘anomalous off’ (Friedel’s law is true) at this step. The default option is to merge the largest cluster with Friedel’s law set to false. During this merging procedure an individual directory containing *XSCALE* input and output files is created. This directory also contains a script for running the program *POINTLESS*, to merge selected datasets in mtz format. In addition, it contains a picture in portable network graphics (.png) format of the dendrogram as a reminder of the clustering threshold.

HCA can be performed with *ccCluster* from the command line, by calling the command with the (-p) option. This way of using the program allows its integration into pipelines for fully automated structure solution, which requires the merging of diffraction data collected from many crystals of the same target. In order to do so, the linkage threshold that is automatically estimated by *ccCluster* must, at the very least, lead to a highly complete dataset. This can be achieved by running *ccCluster* with the (-m) option which calls the minimal threshold for completeness routine.

### 2.5. GUI description   

**Figure 2:**
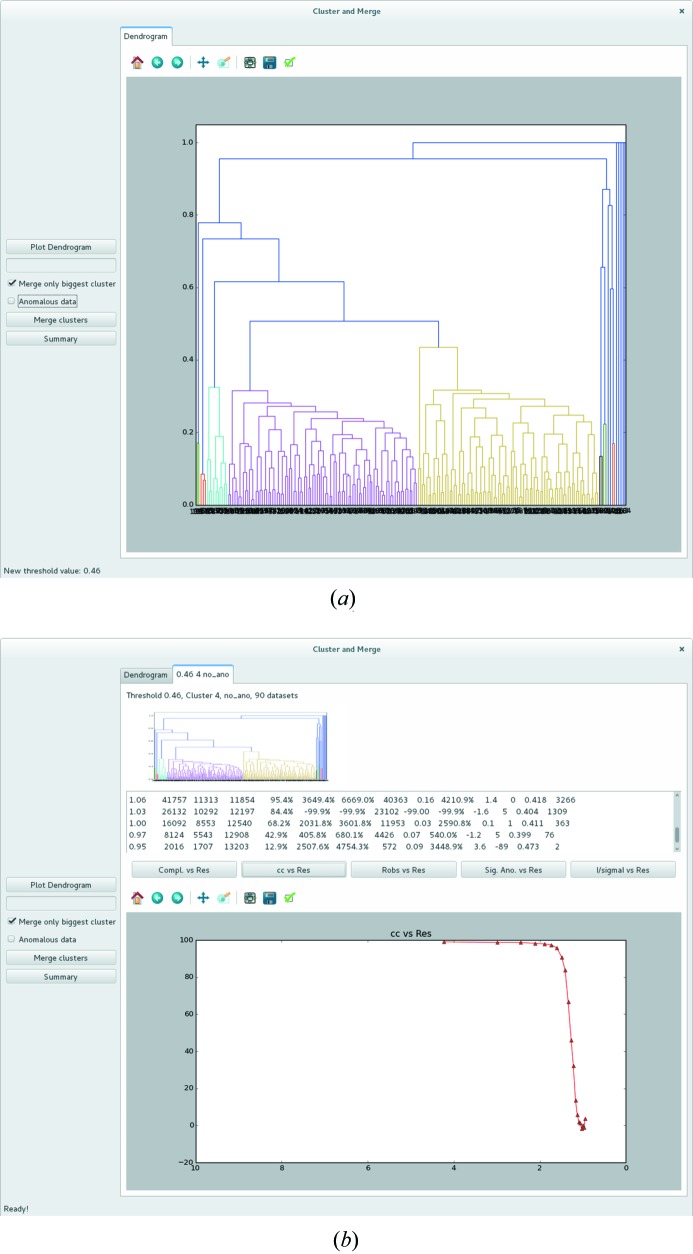
Main features of the *ccCluster* GUI. (*a*) Main panel. The dendrogram is coloured according to the chosen clustering thresholds. Blue branches represent nodes above the thresholds chosen, meaning that they will not be used during the merging step. On the left, buttons allow the user to launch the merging procedure. (*b*) Results panel. A tab is produced for each merged group of datasets, allowing the plotting of statistics calculated using *XSCALE*. Each tab code corresponds to the name of the folder containing the output of merging.

Rapid user interaction is highly desirable when evaluating the effects of choosing different HCA linkage thresholds for partial dataset merging. To this end we have developed a GUI (Fig. 2 ▸) which can be launched after an initial HCA run. The main panel (Fig. 2 ▸ *a*) of the GUI displays the dendrogram itself as well as mouse-clickable buttons for launching the merging procedure and setting/unsetting the ‘anomalous’ flag. Another checkbox allows the choice between merging only the largest cluster at a certain threshold (default) or all clusters below this threshold. The results panel (Fig. 2 ▸ *b*) of the GUI gives the user a quick overview of the quality of merged datasets. Along with a picture of the dendrogram and an extraction of the XSCALE.LP statistics, it is possible to plot the values for CC_1/2_ (Karplus & Diederichs, 2012 ▸), sigAno (|*F* ^+^ − *F* ^−^|/σ) and 〈*I*/σ(*I*)〉 as a function of resolution. Ordering of the different processing steps is conveniently kept by a summary, also shown in the main panel. This gives information about which merged datasets have the better resolution and which have the best CC_1/2_.

## 3. Example of SSX data clustering   

To illustrate the application of *ccCluster* to serial crystallography data, partial datasets, each comprising 2° of diffraction data with an oscillation range of 0.1°, were collected at the ESRF beamline ID29 (De Sanctis *et al.*, 2012 ▸) from 200 micro-crystals (smaller than 20 µm in the largest dimension) of thaumatin contained in a single sample holder. Of the 200 partial datasets collected, 184 were successfully integrated using *XDS* and were then used as input for *ccCluster*. Each dataset contained on average 2483 reflections and had an average overall completeness of 4.9%.

### 3.1. GUI processing and distance definition comparison   

**Figure 3:**
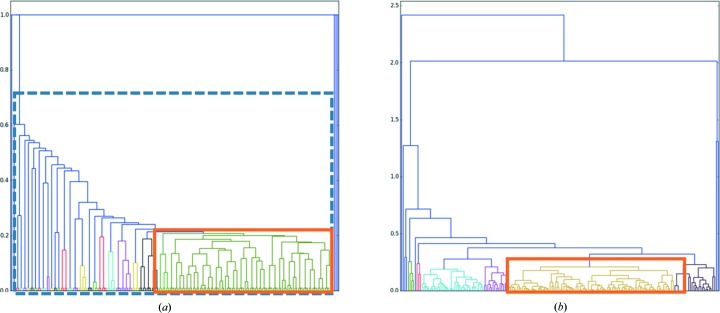
Dendrograms representing the clustering of 184 2° wedges collected from different thaumatin crystals. (*a*) Clustering according to correlation coefficient. The orange rectangle represents the cluster at a threshold of 0.21 and the blue dashed rectangle the cluster at 0.8. (*b*) Clustering based on variation of unit-cell parameters. The selected cluster (orange rectangle) comprises 90 datasets at a threshold of 0.27.

Wedges containing only 2° of diffraction data present a rather difficult case for cluster analysis. The unit-cell parameters cannot be determined with sufficient precision and the calculation of intensity-based correlation coefficients is adversely affected by the low number of common reflections between each wedge. To test the performance of both approaches, two HCA runs were carried out: one using intensity-based correlation coefficients, the other based on variation of unit-cell dimensions. For HCA using cc_(*a*,*b*)_, automatic analysis in *ccCluster* suggested the merging of 123 datasets clustering at a linkage distance of 0.25, with subsequent visual analysis of the dendrogram *via* the *ccCluster* GUI suggesting the merging of partial datasets from a smaller cluster (98 datasets) with a linkage distance of 0.21 (Fig. 3 ▸). The partial datasets in the smaller cluster were thus merged and scaled (Table 1 ▸). Subsequently structure solution was carried out using molecular replacement in *DIMPLE* (http://ccp4.github.io/dimple/) and model refinement (Table 1 ▸) effected with iterative cycles of *REFMAC* (Murshudov *et al.*, 2011 ▸) and *COOT* (Emsley *et al.*, 2010 ▸). For comparison, we also scaled and merged 179 datasets clustering at a much higher linkage distance of 0.8 (Table 1 ▸) and used the resulting dataset for structure solution and refinement (Table 1 ▸). HCA using variation of unit-cell dimensions presented a clear distinction between partial dataset subgroups (Fig. 3 ▸ *b*). In this case, the automatic threshold (0.27) suggested by *ccCluster* led to the merging and scaling of 90 partial datasets (Table 1 ▸), with the final dataset also used for structure determination and refinement as outlined above.

As can be seen from Table 1 ▸, all the final datasets allowed successful structure solution and refinement. As might be expected, choosing which partial datasets to merge using HCA based on either cc_(*a*,*b*)_ or variation of unit-cell dimensions produced both better quality datasets and better final refined models than merging partial datasets indiscriminately. However, it is also clear from Table 1 ▸ that both dataset and final refined model quality are better when the choice of partial dataset merging is directed by HCA based on cc_(*a*,*b*)_ than they are when HCA is based on variation of unit-cell dimensions.

For the ensemble of partial datasets described above, running *ccCluster* with the ‘minimal threshold for completeness’ option results in a linkage threshold estimation of 0.2, very close to the 0.21 chosen from manual inspection of the dendrogram. This threshold choice resulted in the merging of 92 datasets, producing a final dataset with almost identical characteristics to that produced by visual inspection of the dendrogram (Table 1 ▸).

**Figure 4:**
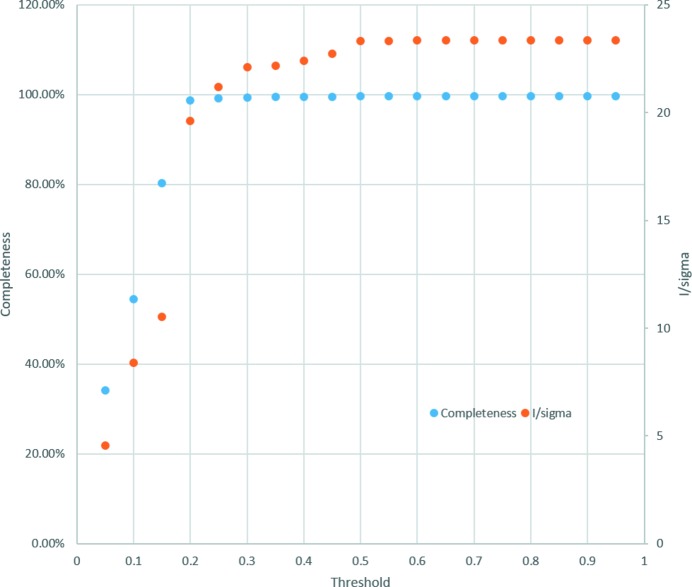
Use of *ccCluster* using the -m option and 184 2° wedges collected from different thaumatin crystals. Here the minimal threshold for 98% completeness is estimated to be 0.2. As outlined in the main text, merging of partial datasets clustering at linkage distances higher than 0.2 results in compiled datasets with slightly higher 〈*I*/σ(*I*)〉, probably because of the increased multiplicity of the final datasets. However, there is no improvement in this metric above a linkage threshold of ∼0.5 as the inclusion of non-isomorphous datasets begins to have an adverse effect on data quality.

To evaluate the efficiency of the -m option, *ccCluster* was used, employing the -t command line option, to merge partial datasets clustering at various linkage threshold levels, ranging from 0.05 to 1.0 in steps of 0.05. The results of this exercise are shown in Fig. 4 ▸. As can be seen, ∼100% completeness of the resulting dataset is achieved only when the linkage distance used is 0.2 or above. As might be expected, merging partial datasets clustering at linkage distances higher than 0.2 results in compiled datasets with slightly higher 〈*I*/σ(*I*)〉, probably due to the increased multiplicity of the final datasets. However, even here there is no improvement in 〈*I*/σ(*I*)〉 above a linkage threshold of ∼0.5 as the inclusion of non-isomorphous datasets begins to have an adverse effect on data quality.

## 4. Application to data from a sulfur-SAD experiment   

**Figure 5:**
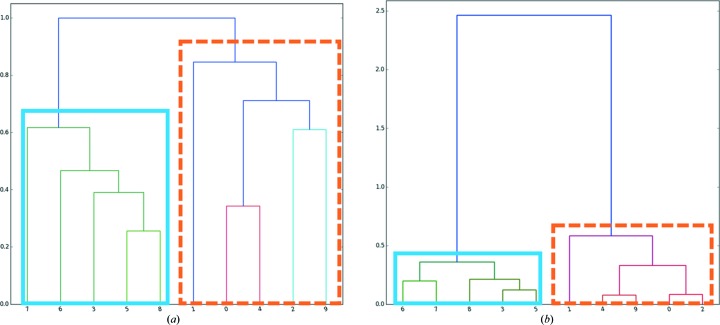
Dendrograms from HCA on nine datasets collected for lysozyme S-SAD. (*a*) Dendrogam obtained by clustering according to correlation coefficients. (*b*) Dendrogam obtained by clustering according to unit-cell variation. In both cases one finds two clusters containing the same datasets.

**Figure 6:**
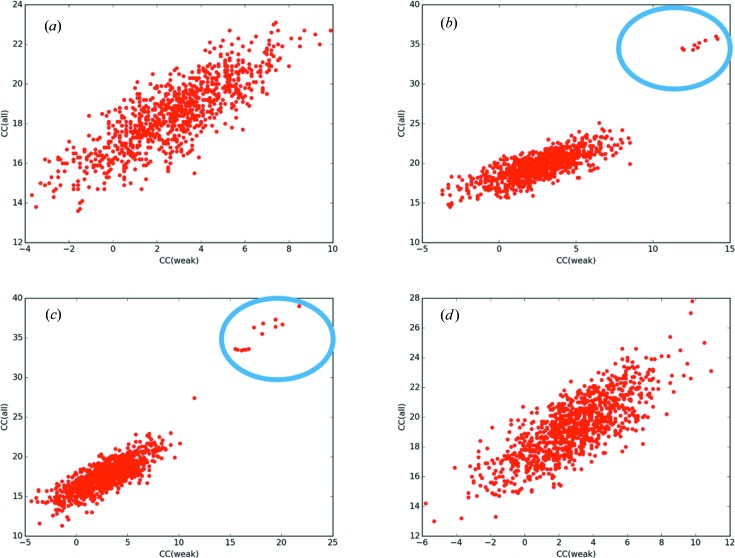
Sub-structure determination results for different clusters from a lysozyme sulfur-SAD experiment based on nine individual datasets. Plots were produced from the results of *SHELXD* (Sheldrick, 2010 ▸) run through the interface *hkl2map* (Pape & Schneider, 2004 ▸). (*a*) *SHELXD* CC(weak) *versus* CC(all) plots produced for one randomly chosen dataset. No solution is found in this case. (*b*) *SHELXD* CC(weak) *versus* CC(all) plots for the dataset produced by merging datasets clustering at a threshold of 0.8. Clear solutions are present. (*c*) *SHELXD* CC(weak) *versus* CC(all) plots for the dataset produced by merging datasets clustering at a threshold of 0.64. Again clear solutions are present. (*d*) *SHELXD* CC(weak) *versus* CC(all) plots for the dataset produced by merging all nine datasets together. No clear solution is found in this case.

The application of *ccCluster* described above concerns the use of HCA to compile a complete dataset from small wedges of data collected from many different crystals. While this is the main intended application of *ccCluster*, the program is also clearly applicable to the HCA of complete datasets collected from different crystals of the same target. An example of such a use of *ccCluster* is in the compilation of high-multiplicity datasets such as those required in S-SAD experiments (Olieric *et al.*, 2016 ▸). Fig. 5 ▸ shows the HCA [cc_(*a*,*b*)_], using *ccCluster*, of nine individual datasets (supporting information, Table S1) collected from crystals of tetragonal lysozyme using X-rays of λ = 2.0 Å at ESRF beamline ID29. Here, none of the individual datasets could be used for successful S-SAD structure determination using default parameters in *hkl2map* (Pape & Schneider, 2004 ▸) (Fig. 6 ▸ *a*) nor could a dataset compiled by merging all nine datasets (Fig. 6 ▸ *d*). The *ccCluster* HCA dendrogram shows that the datasets can be split into two groups of 5 and 4 datasets, respectively, one at a linkage threshold of 0.64 (Fig. 6 ▸ *c*) and another at a threshold of 0.83 (Fig. 6 ▸ *b*). Complete datasets were thus generated by the merging of the datasets in each of these two clusters (Table 2 ▸), and these were used in the automated SAD pipeline *crank2* (Skubák & Pannu, 2013 ▸), with successful structure determination achieved using both datasets. However, they produced slight differences in the completeness of the final model that could be built automatically.

As a comparison, we also performed cluster analysis based on unit-cell parameters, for which the dendrogram is shown in Fig. 5 ▸(*b*). We can observe how one obtains the same two clusters containing the same datasets, thus leading to identical results in the phasing process. Thus, for this case the fact that the clustering is based on the unit-cell variation or the correlation coefficient does not make any significant difference to the results obtained.

In this example, the best results for SAD structure solution are obtained with the cluster with the linkage threshold value 0.64 (Fig. 5 ▸ *a*). It may seem counterintuitive that merging datasets with cc_(*a*,*b*)_ as low as 77% (equivalent to a linkage threshold of 0.64) could improve the anomalous signal required for SAD structure solution. However, the cc_(*a*,*b*)_ used in *ccCluster* is calculated over the whole common resolution range of the datasets collected, and the HCA linkage distances obtained could be dominated by the higher-resolution data shells. Indeed, if we limit our analysis of these S-SAD datasets to a common resolution of 2.5 Å (see supporting information, Fig. S3) the linkage HCA distance for the main cluster drops to ∼0.32, corresponding to 〈cc_(*a*,*b*)_〉 of ∼94%. This shows that at intermediate resolution the datasets in this cluster are more similar to each other than is suggested by including the whole common resolution range in cc-based HCA. As it is usually lower-resolution data that are used to kick-start SAD structure solution processes, this clearly explains why merging of the five datasets in this cluster makes structure solution much more straightforward and suggests that for SAD structure solution protocols exploiting multi-crystal data collection the use of HCA to guide the compilation of final datasets should perhaps best be carried out at resolutions significantly lower than the maximum resolution obtained.

## 5. Conclusions   

Here we have presented *ccCluster*, a software aimed at facilitating the application of HCA in MX experiments. We are confident that the user-friendliness of *ccCluster*, in particular in its GUI mode of operation, will lead to increased and more successful use of HCA in multi-crystal MX. While we have presented two examples as to how *ccCluster* can be used to rapidly perform HCA, to present results and to compile complete datasets, a detailed analysis of the applicability of HCA in multi-crystal MX is clearly beyond the scope of this article and we refer readers to earlier discussions in this regard (Giordano *et al.*, 2012 ▸; Foadi *et al.*, 2013 ▸; Zander *et al.*, 2016 ▸, 2015 ▸). This software has already been installed at the ESRF MX beamlines and used within the context of the SSX BAG for one year. Successful applications have already been published (Zander *et al.*, 2015 ▸, 2016 ▸; Melnikov *et al.*, 2017 ▸).

## Supplementary Material

Supporting information file. DOI: 10.1107/S1600576717015229/ap5019sup1.pdf

## Figures and Tables

**Table 1:** Statistics of serial crystallography experiments Data in parentheses are for the highest-resolution shell.

	Clustering by cc		Clustering by unit cell
Data collection and processing	Threshold 0.21	Threshold 0.8	Threshold 0.27
Wavelength (Å)	2.8	2.8	2.8
Space group	*P*4_2_2_1_2	*P*4_1_2_1_2	*P*4_1_2_1_2
Unit cell (*a*, *b*, *c*) (Å)	58.07, 58.07, 150.56	58.09, 58.09, 150.58	58.04, 58.04, 150.51
Resolution range (Å)	19.81–1.8 (1.85–1.8)	19.67–1.8 (1.83-1.8)	19.80–1.80 (1.85–1.80)
Total No. of reflections	246 000	452 818	250 041
No. of unique reflections	24 532	24 856	24 508
Completeness (%)	98.7 (84.3)	98.6 (78.2)	97.8 (71.0)
Multiplicity	10.0 (2.4)	18.2 (3.8)	10.2 (2.6)
Half-set correlation CC_1/2_	0.997 (0.843)	0.775 (0.528)	0.951 (0.442)
〈*I*/σ(*I*)〉	16.9 (3.8)	17.3 (3.0)	16.5 (3.3)
*R*_pim_	0.029 (0.195)	0.104 (0.329)	0.044 (0.286)
*R*_meas_	0.097 (0.351)	0.357 (0.708)	0.144 (0.508)
*B* factor, Wilson plot (Å^2^)	12.3	25.1	15.1
Final *R*_cryst_	0.144	0.279	0.195
Final *R*	0.175	0.293	0.227

**Table 2:** Statistics for different clustering levels on lysozyme S-SAD experimental data Data in parentheses are for the highest-resolution shell.

	Threshold 0.64	Threshold 0.83	Threshold 1.0
Wavelength (Å)	2.0	2.0	2.0
Space group	*P*4_3_2_1_2	*P*4_3_2_1_2	*P*4_3_2_1_2
Unit cell (*a*, *b*, *c*) (Å)	77.38, 77.38, 38.69	78.33, 78.33, 37.80	77.81, 77.81, 38.30
Resolution range (Å)	19.35–2.00 (2.05–2.00)	19.61–2.00 (2.05–2.00)	19.45–1.98 (2.05–2.0)
Total No. of reflections	639 036	480 170	1 254 944
No. of unique reflections	8419	8378	8383
Completeness (%)	99.9 (99.2)	99.8 (98.8)	99.9 (99.9)
Multiplicity	95.2 (26.5)	57.3 (12.6)	149.7 (38.9)
Half-set correlation CC_1/2_	1.000 (0.999)	0.996 (0.954)	0.998 (0.984)
〈*I*/σ(*I*)〉	55.9 (14.0)	32.7 (8.4)	22.2 (6.0)
*R*_pim_	0.009 (0.039)	0.026 (0.066)	0.047 (0.080)
*R*_meas_	0.093 (0.208)	0.149(0.172)	0.446 (0.493)
*B* factor, Wilson plot (Å^2^)	18.0	14.2	25.1
Mid-slope of anomalous normal probability†	1.59	1.12	0.701
CC_ano_ ‡	0.63	0.54	0.53
Correct solutions per 1000 trials	12	9	0
CC_weak_/CC_all_ of best solution	21.9/39.08	14.6/35.8	10.5/25.02

† As calculated by *AIMLESS* (Evans & Murshudov, 2013 ▸).

‡ Calculated at 2 Å resolution.
