# Supplementary material for: Hierarchical clustering for multiple-crystal macromolecular crystallography experiments: the ccCluster program
Source: J Appl Crystallogr. 2017 Nov 29;50(Pt 6):1844–51. doi: 10.1107/S1600576717015229 (PMC5713145; doi:10.1107/S1600576717015229)
Supplement: Supplementary file 1 [file j-50-01844-sup1.pdf]

JOURNAL OF
APPLIED
CRYSTALLOGRAPHY

**Volume 50 (2017)**

**Supporting information for article:**

# Hierarchical clustering for multiple crystal macromolecular crystallography experiments: the ccCluster program

**Gianluca Santoni, Ulrich Zander, Christoph Mueller-Dieckmann, Gordon Leonard and Alexander Popov**

# Hierarchical clustering for multiple crystal macromolecular crystallography experiments: the ccCluster program.

Gianluca Santoni[a], Ulrich Zander[a,b], Christoph Mueller-Dieckmann[a], Gordon Leonard[a], Alexander Popov[a]

a) Structural Biology Group, European Synchrotron Radiation Facility, 71 Avenue des Martyrs, 38000 Grenoble, France,
b) EMBL Grenoble, 71 Avenue des Martyrs, CS 90181, 38042 Grenoble CEDEX 9, France

*Table S1: Statistics for data collection and analysis parameters for the 9 individual datasets used in the multi-crystal S-SAD experiment discussed in the main text. Dose is calculated with RADDOSE-3D* (Zeldin et al., 2013) *† in the highest resolution shell*

| | 0 | 1 | 2 | 3 | 4 | 5 | 6 | 7 | 8 |
|---|---|---|---|---|---|---|---|---|---|
| Dose (MGy) | 0.5 | 0.2 | 0.5 | 0.3 | 0.5 | 0.2 | 0.5 | 0.2 | 0.1 |
| Unit Cell (*a*,*b*,*c*) (Å): | 78.01, 78.01, 37.88 | 78.48, 78.48, 37.71 | 77.25, 77.25, 38.77 | 77.34, 77.34, 38.78 | 77.35, 77.35, 38.64 | 77.5, 77.5, 38.63 | 77.46, 77.46, 38.76 | 78.45, 78.45, 37.82 | 78.51, 78.51, 37.82 |
| Resolution range (Å): | 39.01 - 2.3 | 39.24 - 2.0 | 38.7 - 2.0 | 38.8 - 2.0 | 38.6 - 1.99 | 38.75 - 2.0 | 38.76 - 2.0 | 39.2 - 2.0 | 39.2- 1.9 |
| Total No. of reflections: | 110976 | 164672 | 163565 | 162701 | 157180 | 152519 | 166135 | 163334 | 155736 |
| No. of unique reflections: | 5611 | 8824 | 8136 | 8398 | 8294 | 8088 | 8330 | 8316 | 8314 |
| Completeness (%): | 98.9 (93.1) | 97.9 (87.1) | 96.8(88.6) | 99.1 (94.5) | 97.8 (86.0) | 96.1 (76.3) | 99.2 (95.0) | 98.6 (91.0) | 97. 2(90.0) |
| Half-set correlation $CC_{1/2}$: (%) | 99.9 (91.7) | 100.0 (99.4) | 100 (99.6) | 99.9 (98.9) | 99.9 (97.7) | 100 (95.1) | 99.9 (99.4) | 100 (98.8) | 99.7 (88.8) |
| $<I/\sigma(I)>$: | 23.6 (2.3) | 50.9 (14.5) | 60.6 (18.5) | 40.3 (10.2) | 29.8 (7.7) | 34.4 (4.4) | 38.2 (12.7) | 41.8 (7.9) | 30.5 (2.3) |
| $R_{meas}$: | 9.1(54.0) | 0.045(0.11) | 0.039 (0.088) | 0.057(0.147) | 0.08 (0.21) | 0.06(0.29) | 0.064(0.10) | 0.052(0.18) | 0.092(0.52) |
| $R_{pim}$ | 0.02(0.17) | 0.01(0.04) | 0.011(0.044) | 0.012(0.058) | 0.016(0.083) | 0.013(0.130) | 0.014(0.039) | 0.011(0.072) | 0.033(0.257) |
| $CC_{ano}$ † | -0.08 | 0.11 | 0.16 | 0.08 | -0.02 | -0.07 | 0.11 | 0.12 | 0.02 |
| Correct solutions per 1000 tials | 0 | 1 | 0 | 0 | 0 | 0 | 0 | 0 | 0 |
| $Cc_{weak}/cc_{all}$ of best solution | 11.5 / 31.7 | 16.8 / 34.6 | 8.5 / 21.9 | 9.6 / 24.3 | 9.9/23.3 | 11.1/24.7 | 8.6 / 21.8 | 10.4 / 24.8 | 14.5 / 35.8 |

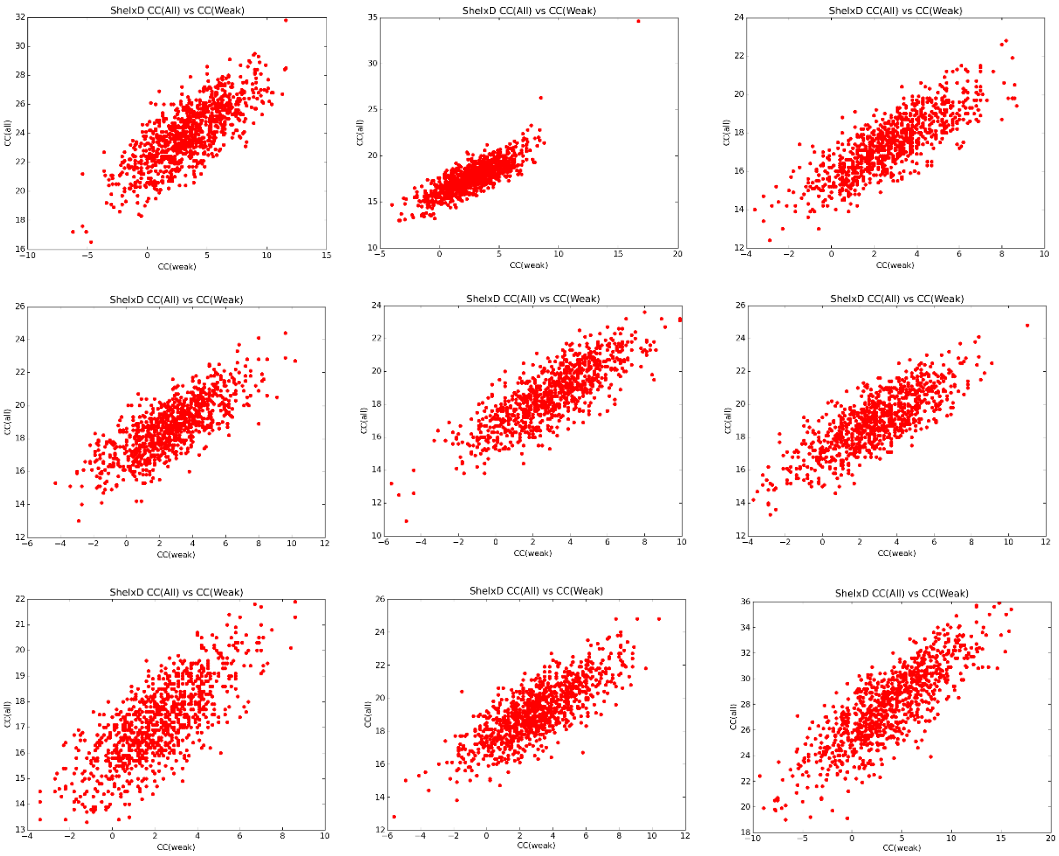


*Figure S1 Sulphur atom substructure determination results for the individual S-SAD data sets from a lysozyme sulfur SAD experiment based on 9 individual datasets. Plots were produced from the results of SHELXD (Sheldrick, 2010) run through the interface hkl2map (Pape & Schneider, 2004).*

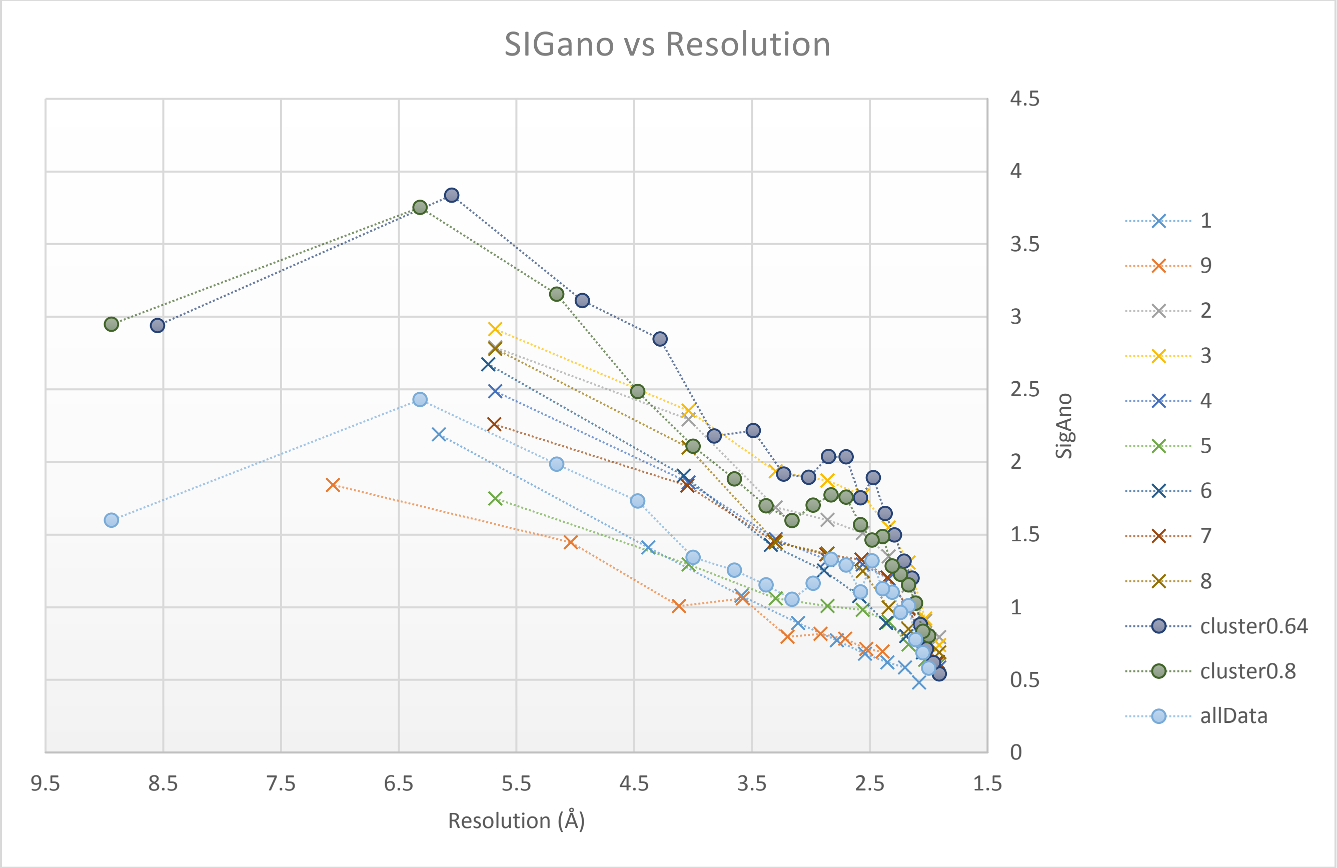


*Figure S2. Plots of anomalous signal (SigAno = <|F(+)-F(-)|/σ>) versus resolution for the S-SAD data sets discussed in the main text.. Crosses represent the individual datasets and dots the three clusters from the paper. We can observe how the two separate clusters lead to improved SigAno values compared to that seen the individual datasets. Merging all of the datasets together leads to a worse SigAno than most of the individual datasets alone.*

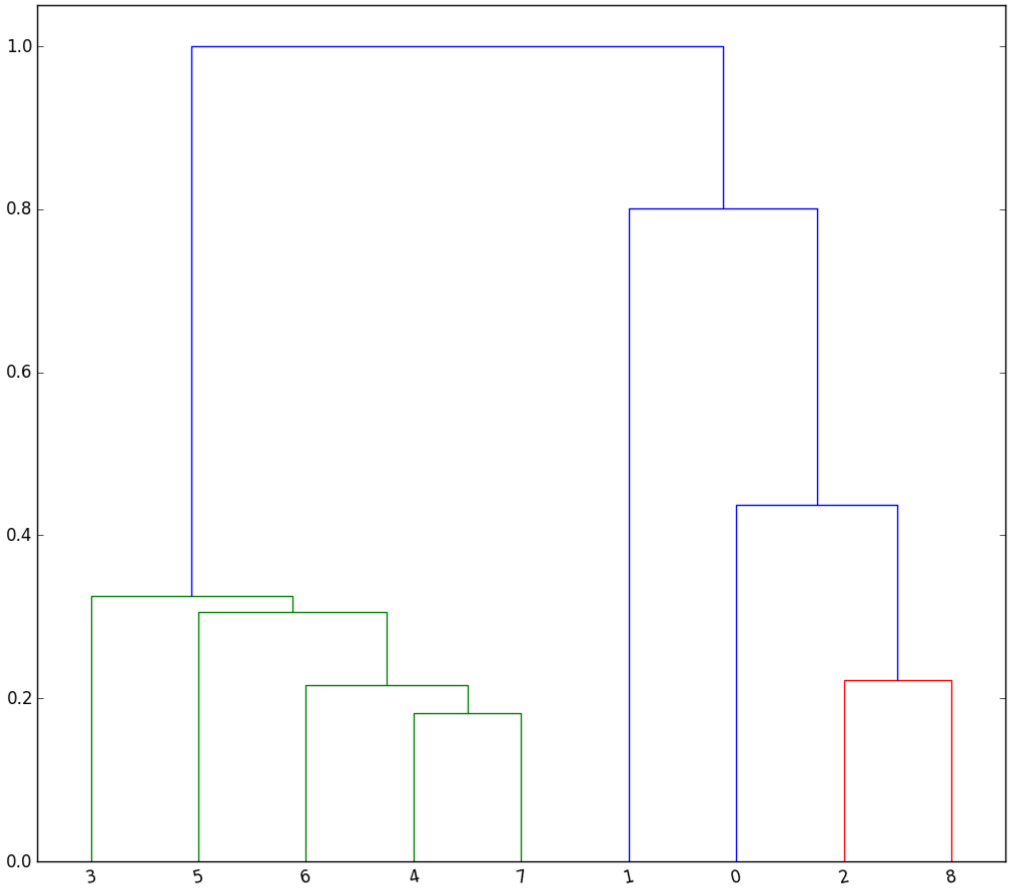


*Figure S3 HCA dendrograms obtained using the 9 S-SAD datasets shown in Table S1 with the analysis limited to a common resolution of 2.5 Å.*
